# Supplementary material for: LRRK2 activation controls the repair of damaged endomembranes in macrophages
Source: EMBO J. 2020 Jul 9;39(18):e104494. doi: 10.15252/embj.2020104494 (PMC7507578; doi:10.15252/embj.2020104494)
Supplement: Supplementary file 1 — Appendix [file EMBJ-39-e104494-s001.pdf]

APPENDIX

TABLE OF CONTENTS

Figure legend for appendix Fig. S1
Appendix figure S1
Appendix table S1
Appendix table S2

Appendix Fig. S1. Imaging controls for Fig. 3.

(A) RAW264.7 macrophages were electroporated with EGFP-Rab8A and stained for Galectin-3, CHMP4B and LC3B. Scale bar = 5 µm.

(B) RAW264.7 macrophages were treated with 1 mM of LLOMe and appearance of LRRK2, Rab8A, CHMP4B, Galectin-3 and LC3B foci over time was visualised by immunofluorescence and high-content imaging. Scale bar = 10 µm.

Appendix Fig. S1

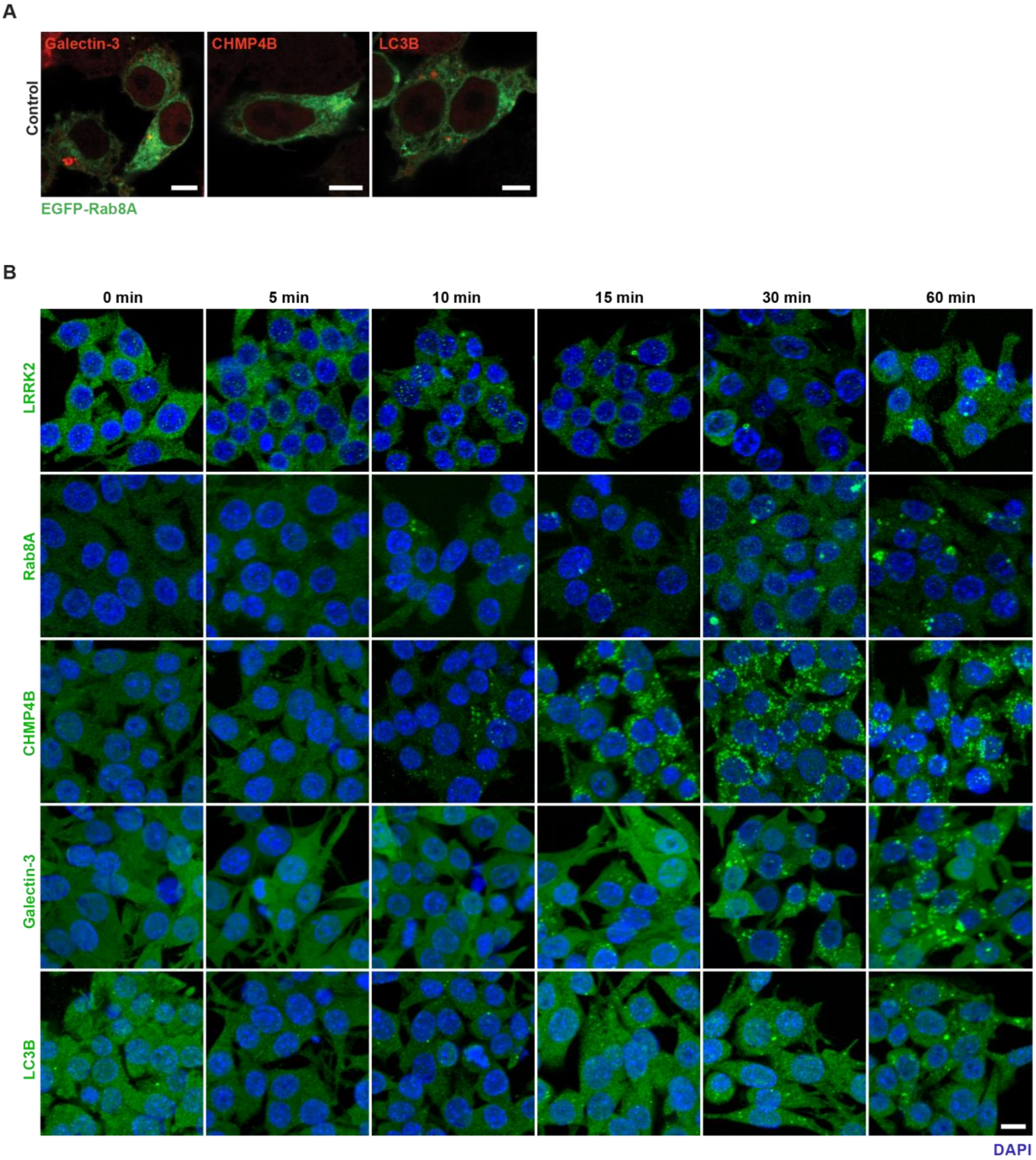

Appendix table S1: Blood donor demographics and clinical information

	Sex	Age at Entry	Age at Onset (AAO)	Disease Duration (years)	Hoehn and Yahr Stage	Mutation
PD_1	M	64.0	56	8.0	2	p.R1441C
PD_2	F	65.4	58	7.4	2	p.G2019S
PD_3	F	66.1	54	12.1	2	p.G2019S
Average (SD)	-	*65.17 (1.07)*	*56.0 (2.0)*	*9.17 (2.56)*	-	-
Control_1	M	64.6	-	-	0	No PD variants detected
Control_2	F	64.7	-	-	0	No PD variants detected
Control_3	M	72.8	-	-	0	No PD variants detected
Average (SD)	-	*67.37 (4.71)*	-	-	-	-

Appendix table S2: Pathogenic Parkinson's Disease variants screened for during genotyping

LRRK2_G2019S	PARK2_E79X	PARK2_E395X	PINK1_A168P
LRRK2_R1441C	PARK2_M192L	PARK2_A398T	PINK1_R246X
LRRK2_Y1699C	PARK2_K211N	PARK2_E409X	PINK1_Y258X
LRRK2_I2020T	PARK2_C212G	PARK2_G429E	PINK1_T313M
PARK2_M1L	PARK2_R234Q	PARK2_G430D	PINK1_W437X
PARK2_A31D	PARK2_V258M	PARK7_L166P	PINK1_G440E
PARK2_R33Q	PARK2_D280N	PINK1_Q456X	SNCA_A30P
PARK2_Q40X	PARK2_C289G	PINK1_R464H	SNCA_E46K
PARK2_R42H	PARK2_Q311H	PINK1_R492X	SNCA_A53T
PARK2_R42P	PARK2_T351P	PINK1_C92F	PARK2_R275W
PARK2_V56E	PARK2_G359D	PINK1_Q129X	VPS35_D620N
