# Supplementary material for: LRRK2 activation controls the repair of damaged endomembranes in macrophages
Source: EMBO J. 2020 Jul 9;39(18):e104494. doi: 10.15252/embj.2020104494 (PMC7507578; doi:10.15252/embj.2020104494)
Supplement: Supplementary file 2 — Expanded View Figures PDF [file EMBJ-39-e104494-s002.pdf]

Expanded View Figures

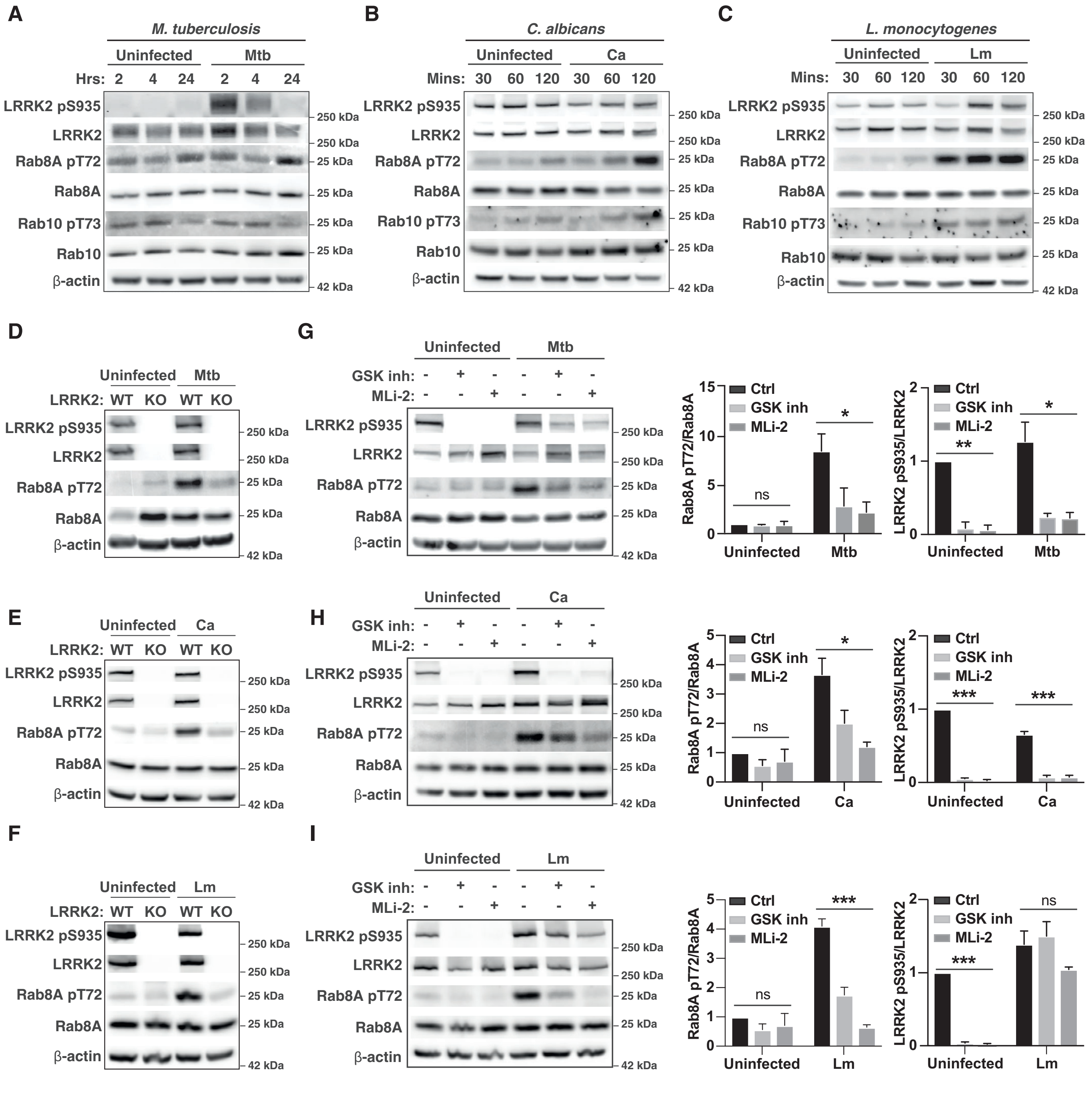

Figure EV1.

Figure EV1. Infection triggers LRRK2-dependent Rab8A phosphorylation in macrophages.

A–C RAW264.7 macrophages were left uninfected or infected with (A) *Mycobacterium tuberculosis* (Mtb), (B) *C. albicans* (Ca) or (C) *L. monocytogenes* (Lm) for the indicated time, and Rab8A pT72, Rab10 pT73 and LRRK2 pS935 levels were analysed by Western blot.

D–F WT and LRRK2 KO macrophages were left uninfected or infected with (D) Mtb for 24 h, (E) Ca or (F) Lm for 120 min. Rab8A pT72 and LRRK2 pS935 phosphorylation was analysed by Western blot.

G–I RAW264.7 macrophages were pre-treated with 1 μM GSK2578215A (GSK inh) or 0.1 μM MLi-2 and left uninfected or infected with (G) Mtb for 24 h, (H) Ca or (I) Lm for 120 min. Rab8A pT72 and LRRK2 pS935 phosphorylation was analysed by Western blot and quantified by densitometry. Beta-actin was used as loading control. Data represent the mean + SEM of three independent biological replicates. One-way ANOVA followed by Dunnett's test against DMSO control. ns = non-significant; $^{*}P < 0.05$; $^{***}P < 0.001$

Source data are available online for this figure.

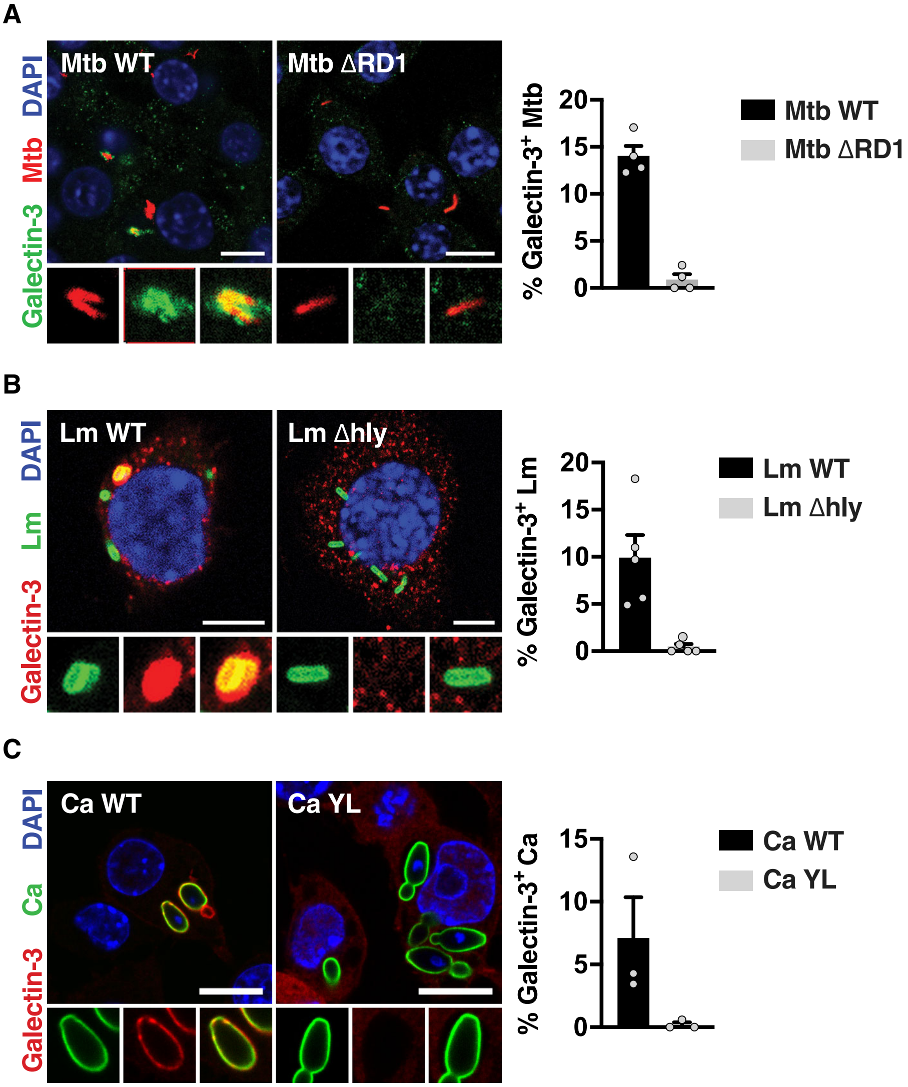

Figure EV2. Galectin-3 recruitment to the pathogens used in this study.

RAW264.7 macrophages were infected with the indicated strains of Mtb, Ca or Lm, and Galectin-3 recruitment was visualised by immunofluorescence. Data represent mean + SEM of three to four independent biological experiments.

A Infection with Mtb WT or Mtb ΔRD1 for 24 h. Scale bar = 10 μm.

B Infection with Lm WT or Lm Δhly for 60 min. Scale bar = 5 μm.

C Infection with Ca WT or Ca YL for 60 min. Scale bar = 10 μm.

 © 2020 The Authors

Figure EV3. LRRK2 mediates Rab8A recruitment to pathogen-containing phagosomes.

A WT and LRRK2 KO macrophages were transfected with EGFP-Rab8A and infected with Mtb for 24 h. Scale bar = 10 μm.

B, C (B) WT and LRRK2 KO macrophages were infected with Ca and (C) Lm for 60 min. Rab8A recruitment was visualised by immunofluorescence. Scale bar = 5 μm.

D Macrophages were transfected with EGFP-Rab8A, treated with 1 μM GSK2578215A (GSK inh) and infected with Mtb for 24 h. Scale bar = 10 μm.

E, F (E) Macrophages were treated with the GSK LRRK2 kinase inhibitor (1 μM) and infected with Ca or (F) Lm for 60 min. Rab8A recruitment was visualised by immunofluorescence. Scale bar = 5 μm.

G–I Representative images of macrophages transfected with EGFP-Rab8A-WT or EGFP-Rab8A-T72A and infected with (G) Mtb for 24 h, (H) Ca for 60 min and (I) Lm for 30 min. Scale bar = 5 μm.

Data information: Right panels show the quantification of the percentage of Rab8A-positive phagosomes. Data represent the mean + SEM of three independent biological replicates. Student's *t*-test; $^*P < 0.05$; $^{**}P < 0.01$; $^{***}P < 0.001$.

© 2020 The Authors

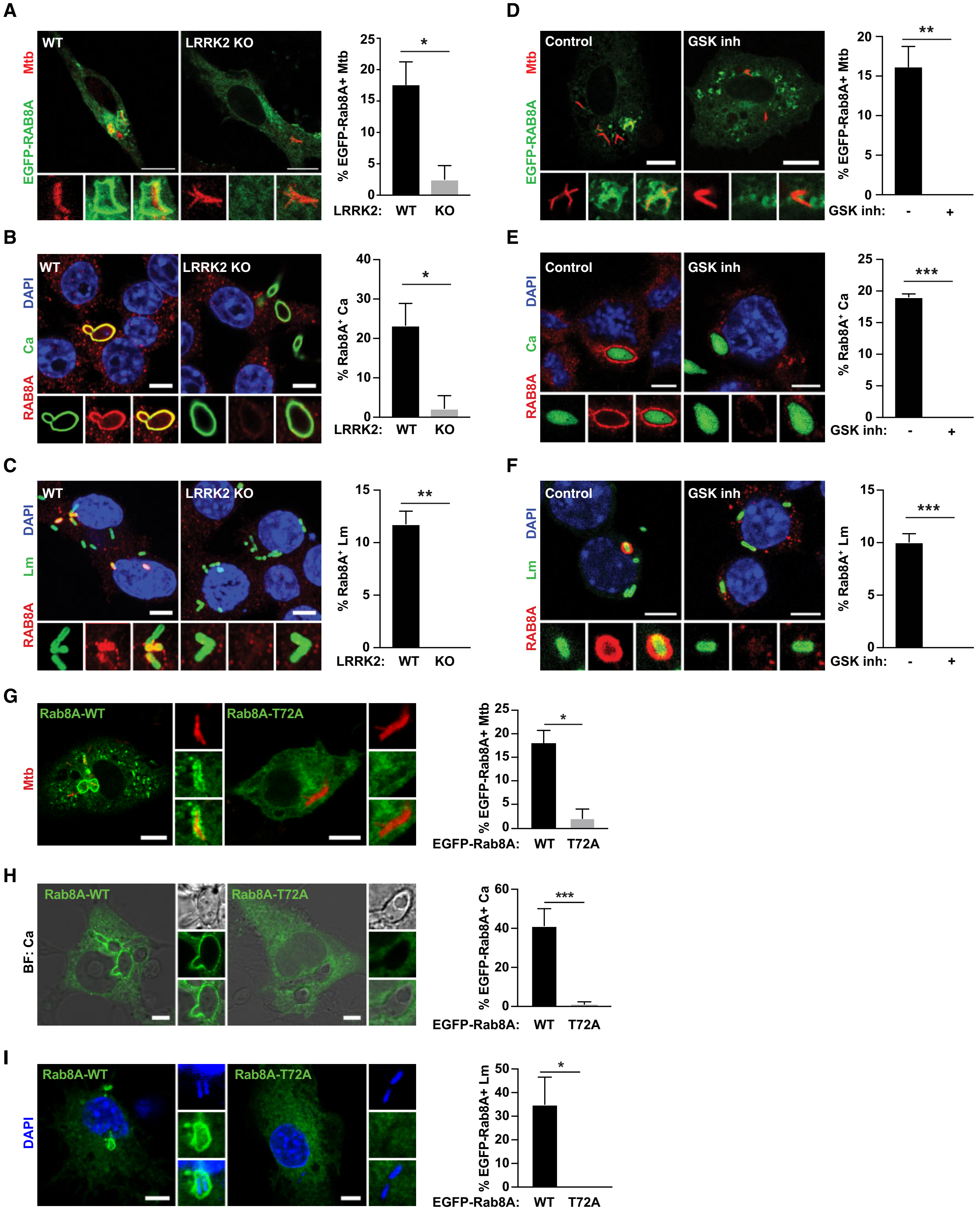

Figure EV3.

© 2020 The Authors

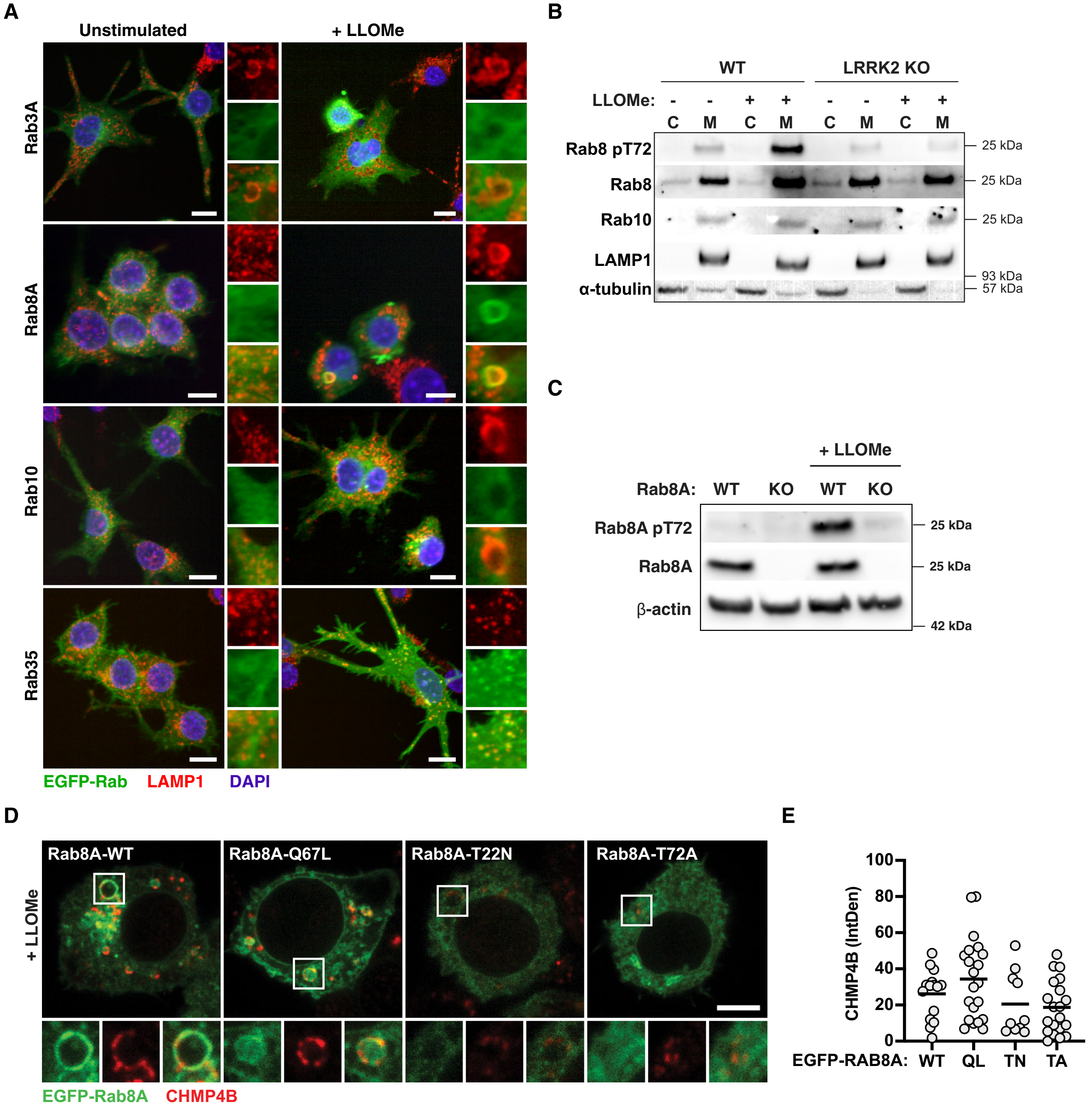

Figure EV4. Characterisation of Rab GTPase behaviour after sterile damage.

A RAW264.7 macrophages were electroporated with EGFP-Rab3A, EGFP-Rab8A, EGFP-Rab10 and EGFP-Rab35. Cells were treated with 1 mM of LLOMe for 30 min, and Rab recruitment to LAMP1 + compartments was monitored by high-content immunofluorescence imaging. Scale bar = 10 μm.

B WT and LRRK2 KO RAW264.7 macrophages were treated with 1 mM LLOMe for 30 min. Cells were separated into cytosolic (C) and membrane (M) fractions and analysed for Rab8A pT72, Rab8A and Rab10 by Western blot.

C WT and Rab8A KO RAW264.7 macrophages were treated with 1 mM LLOMe for 30 min, and Rab8A and Rab8A pT72 levels were analysed by Western blot.

D RAW264.7 macrophages were electroporated with EGFP-Rab8A-WT, EGFP-Rab8A-Q67L, EGFP-Rab8A-T22N and EGFP-Rab8A-T72A. Cells were treated with 1 mM of LLOMe for 30 min, and CHMP4B recruitment was assessed by confocal microscopy. Scale bar = 5 μm.

E CHMP4B integrated fluorescence density was analysed per cell. Data show values from single cells and mean.

Source data are available online for this figure.

© 2020 The Authors

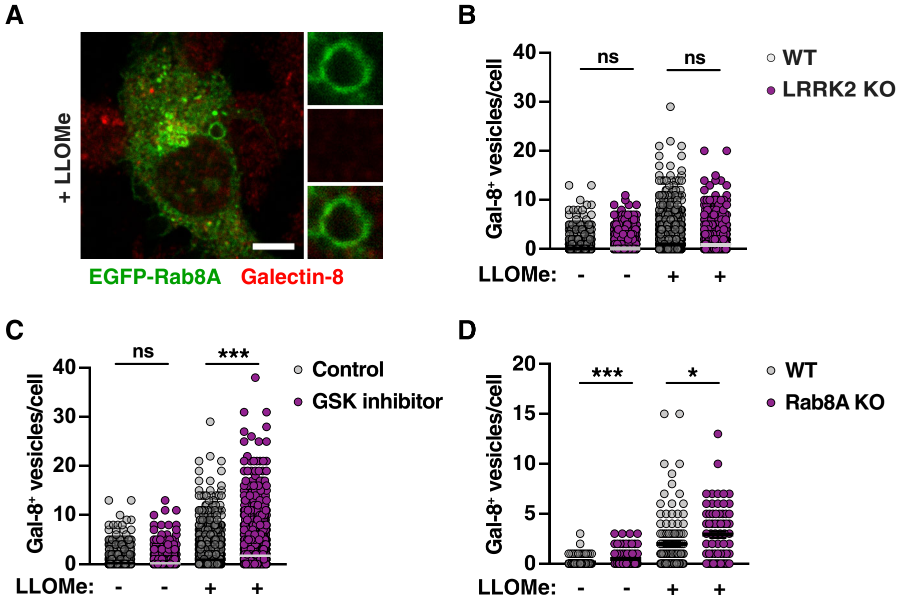

Figure EV5. LRRK2 does not affect sensing of endolysosomal membrane damage by Galectin-8.

A RAW264.7 cells were transfected with EGFP-Rab8A and treated with 1 mM LLOMe for 30 min. Co-localisation with Galectin-8 was visualised by immunofluorescence. Scale bar = 5 µm.

B RAW264.7 WT and LRRK2 KO macrophages were treated with 1 mM LLOMe for 30 min. Galectin-8 vesicle numbers were analysed by immunofluorescence and high-content imaging.

C RAW264.7 macrophages treated or not with 1 µM GSK2578215A (GSK inh) were treated with 1 mM LLOMe for 30 min. Galectin-8 vesicle numbers were analysed by immunofluorescence and high-content imaging.

D RAW264.7 WT or Rab8A KO macrophages were treated with 1 mM LLOMe for 30 min. Galectin-8 vesicle numbers were analysed by immunofluorescence and high-content imaging.

Data information: Data represent number of vesicles per cell. Data show the mean ± SEM of one representative experiment out of three biological replicates shown. ns = non-significant, *P < 0.05, ***P < 0.001 by one-way ANOVA followed by Sidak's multiple comparisons test

© 2020 The Authors
