# Supplementary material for: LRRK2 activation controls the repair of damaged endomembranes in macrophages
Source: EMBO J. 2020 Jul 9;39(18):e104494. doi: 10.15252/embj.2020104494 (PMC7507578; doi:10.15252/embj.2020104494)
Supplement: Supplementary file 3 — Movie EV1 [file EMBJ-39-e104494-s003.zip › Movie EV1_legend.docx]

**Movie EV1:** Time lapse recording corresponding to the image stills shown in figure 5C. RAW264.7 macrophages co-expressing EGFP-Rab8A and RFP-Galectin-3 were treated with 1 mM LLOMe and analysed by live cell imaging.
