# Supplementary figures and images for: LRRK2 activation controls the repair of damaged endomembranes in macrophages
Source: EMBO J. 2020 Jul 9;39(18):e104494. doi: 10.15252/embj.2020104494 (PMC7507578; doi:10.15252/embj.2020104494)

**Figure EV1**

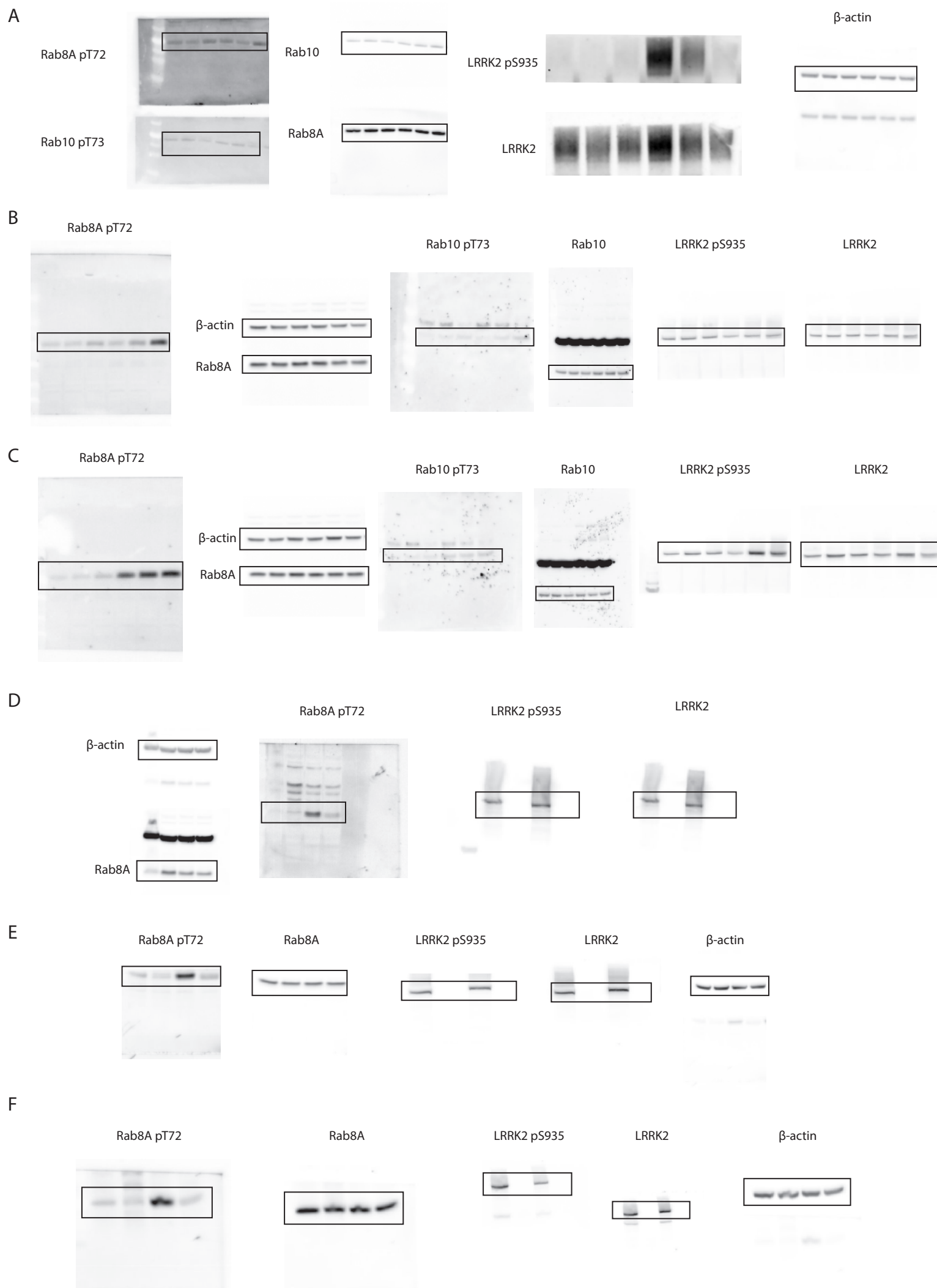

Figure EV1 continued

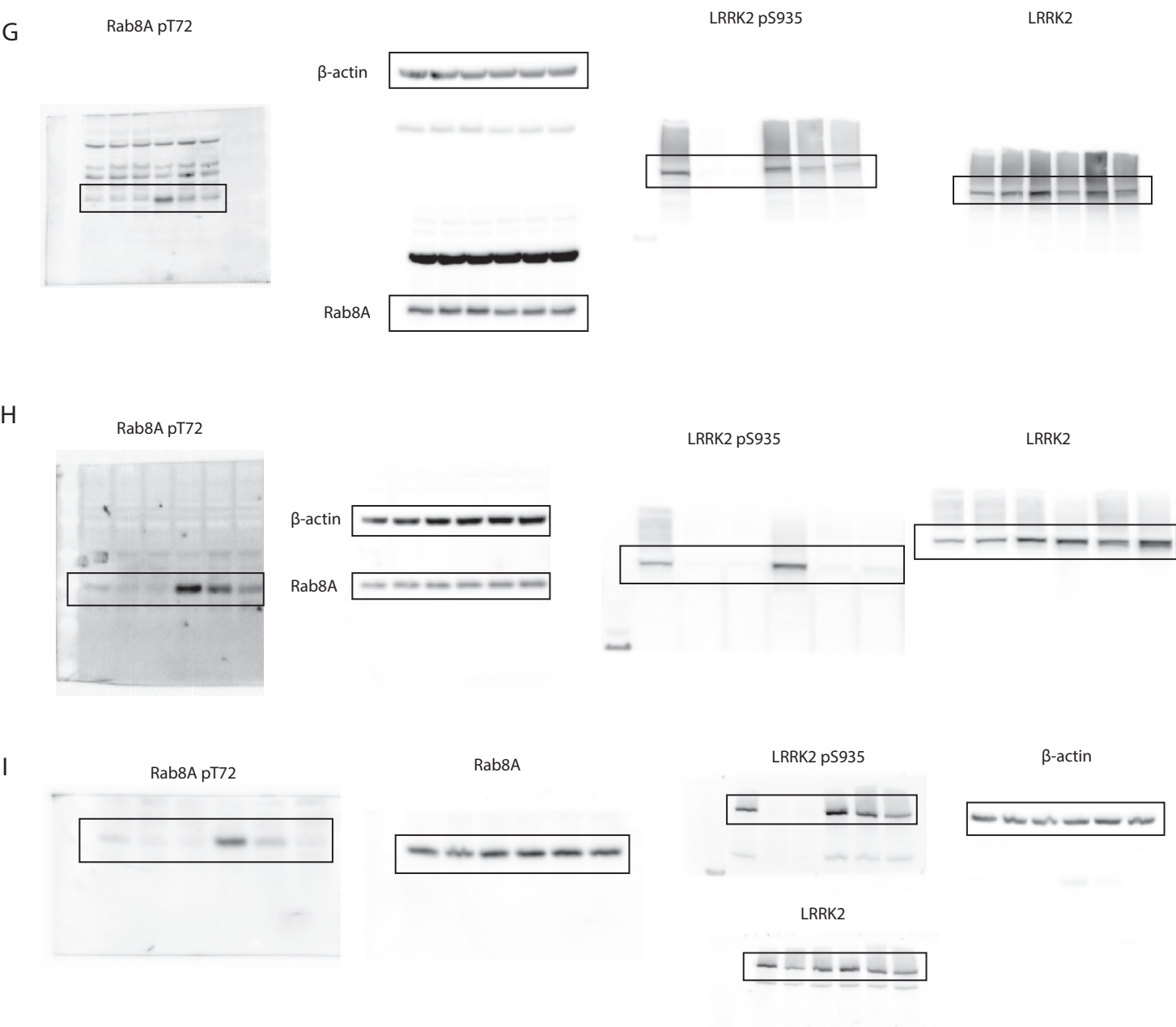

Supplement: Supplementary file 4 — Source Data for Expanded View [file EMBJ-39-e104494-s008.zip › SourceDataFor_FigEV1.pdf]

Figure EV4

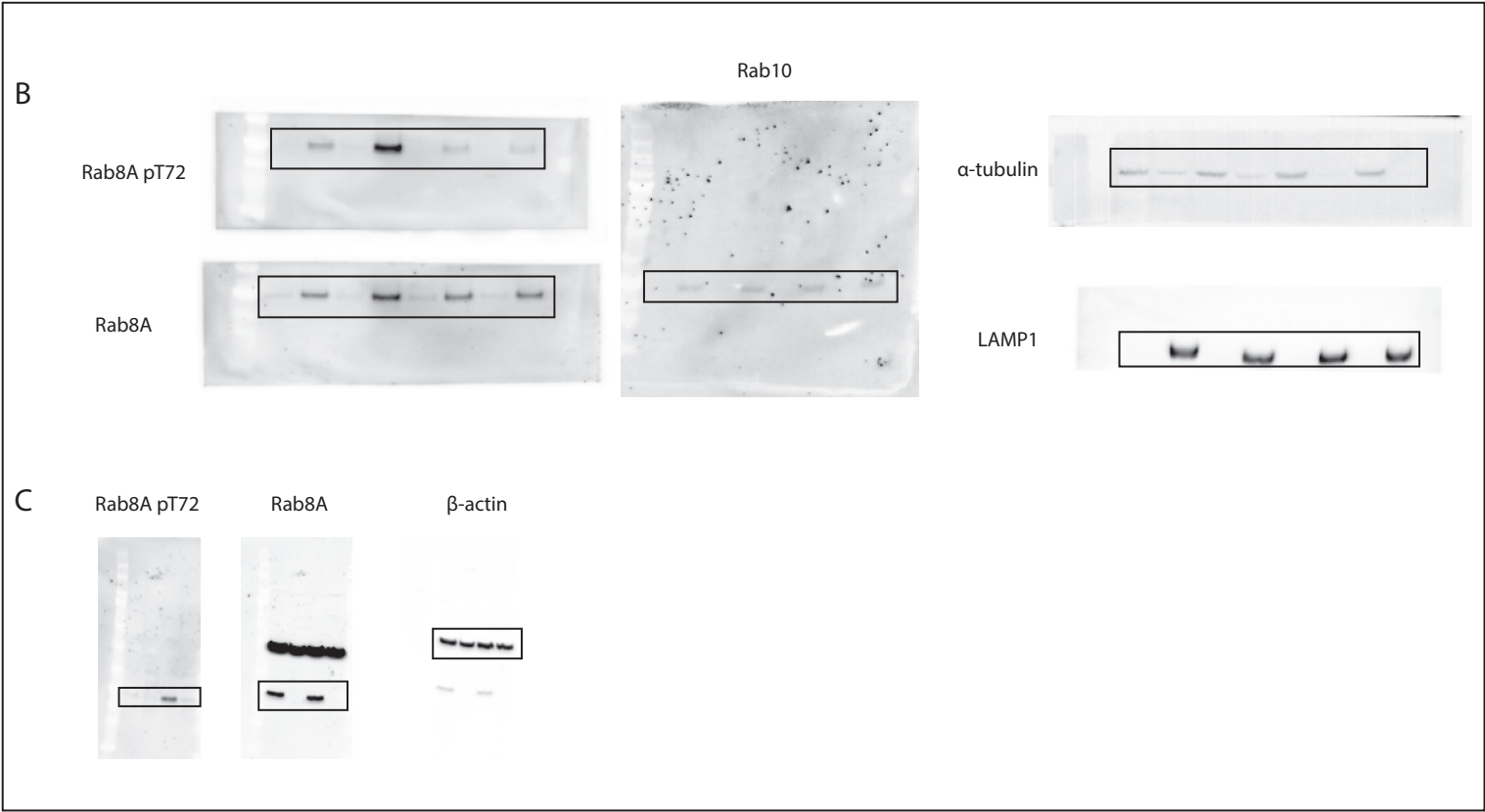

Supplement: Supplementary file 4 — Source Data for Expanded View [file EMBJ-39-e104494-s008.zip › SourceDataFor_FigEV4.pdf]

Figure 1

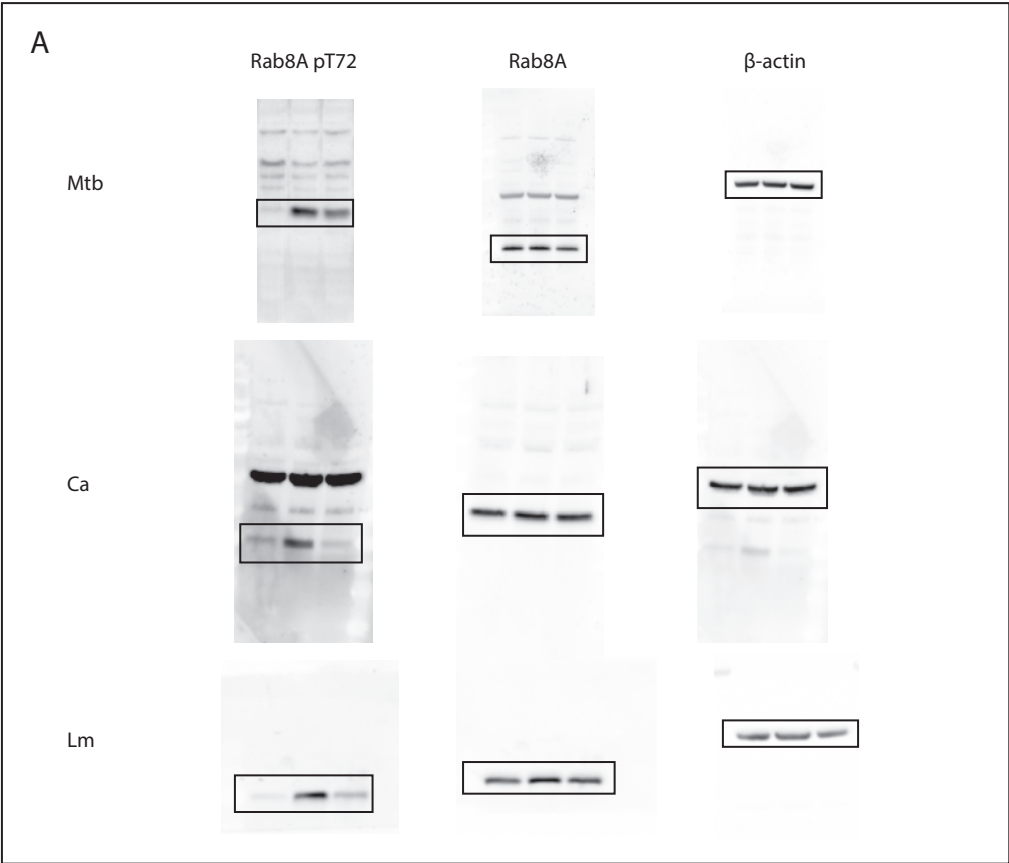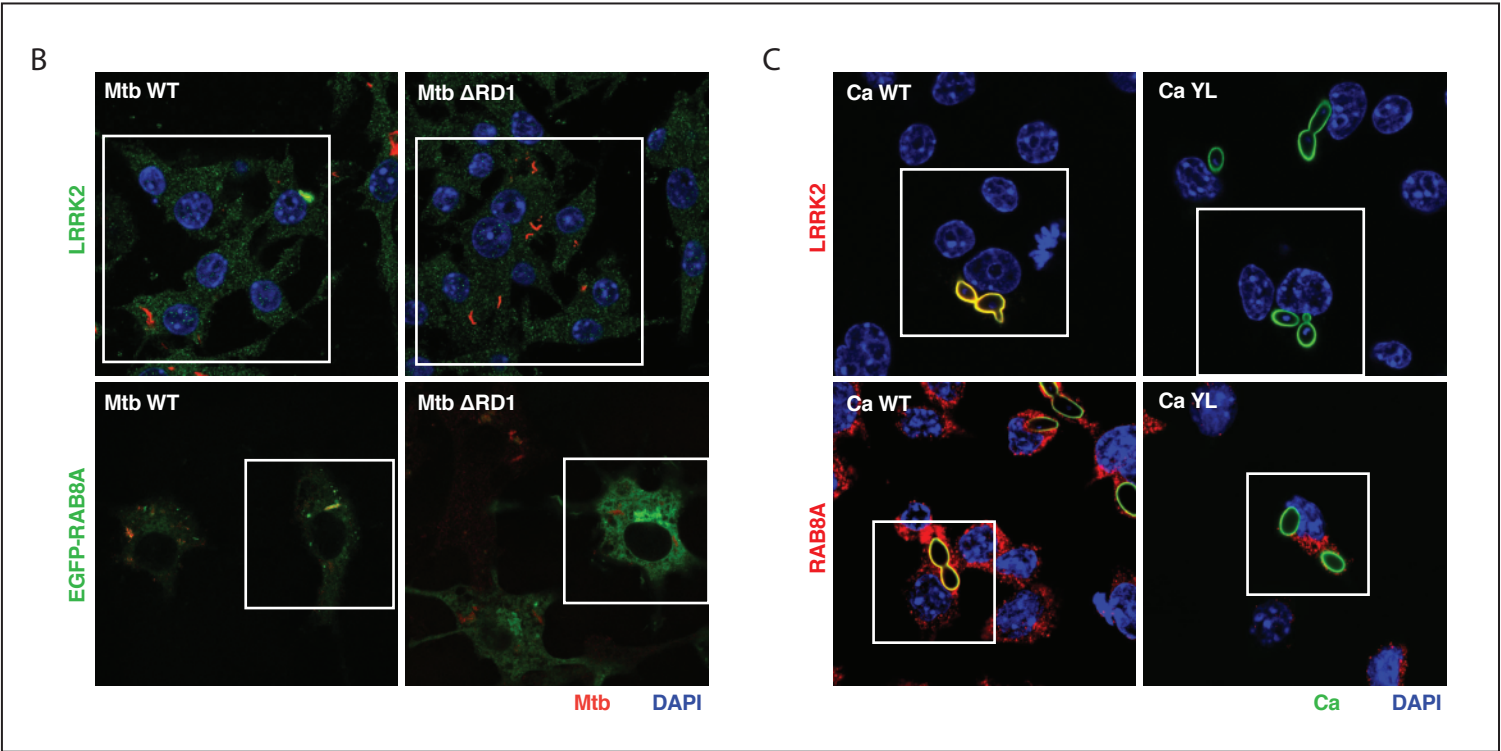

Supplement: Supplementary file 6 — Source Data for Figure 1 [file EMBJ-39-e104494-s004.pdf]

Figure 2

A

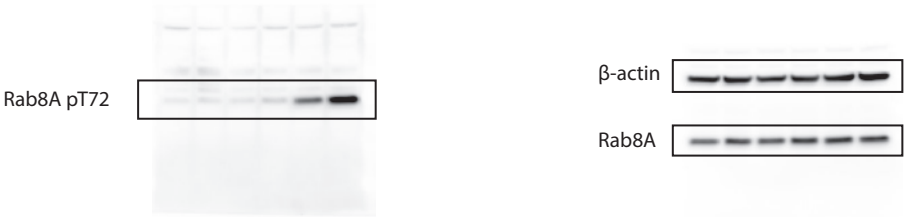

B

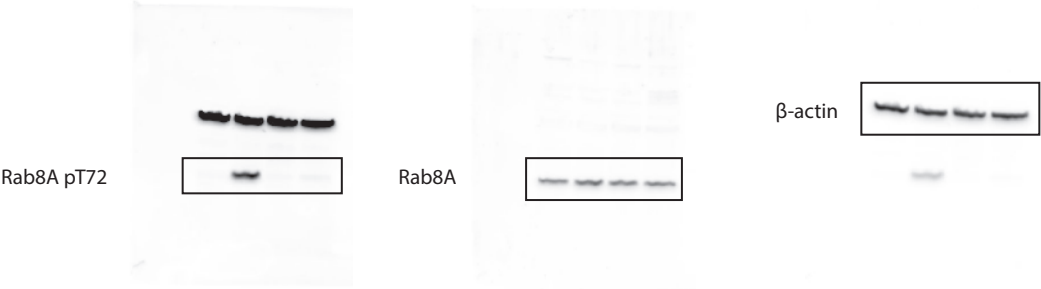

C

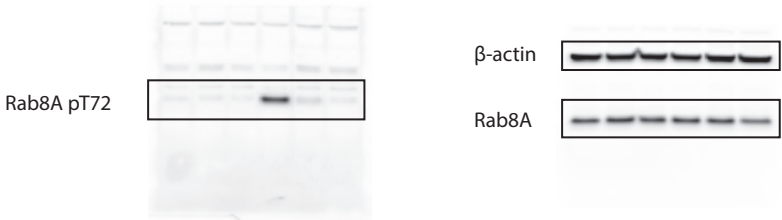

Supplement: Supplementary file 7 — Source Data for Figure 2 [file EMBJ-39-e104494-s005.pdf]

Figure 3E

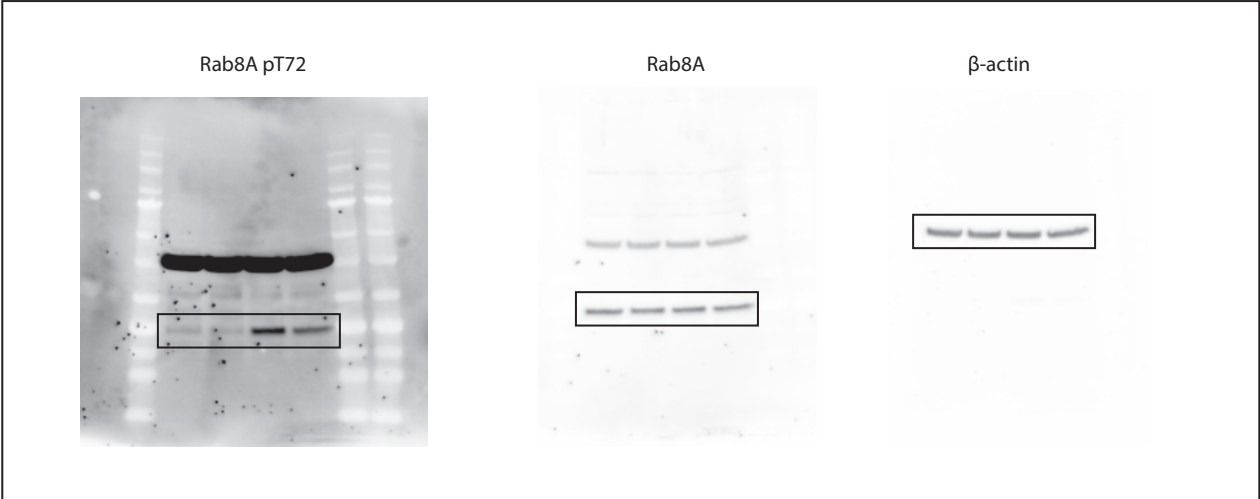

Supplement: Supplementary file 8 — Source Data for Figure 3 [file EMBJ-39-e104494-s006.pdf]

**Figure 7A**

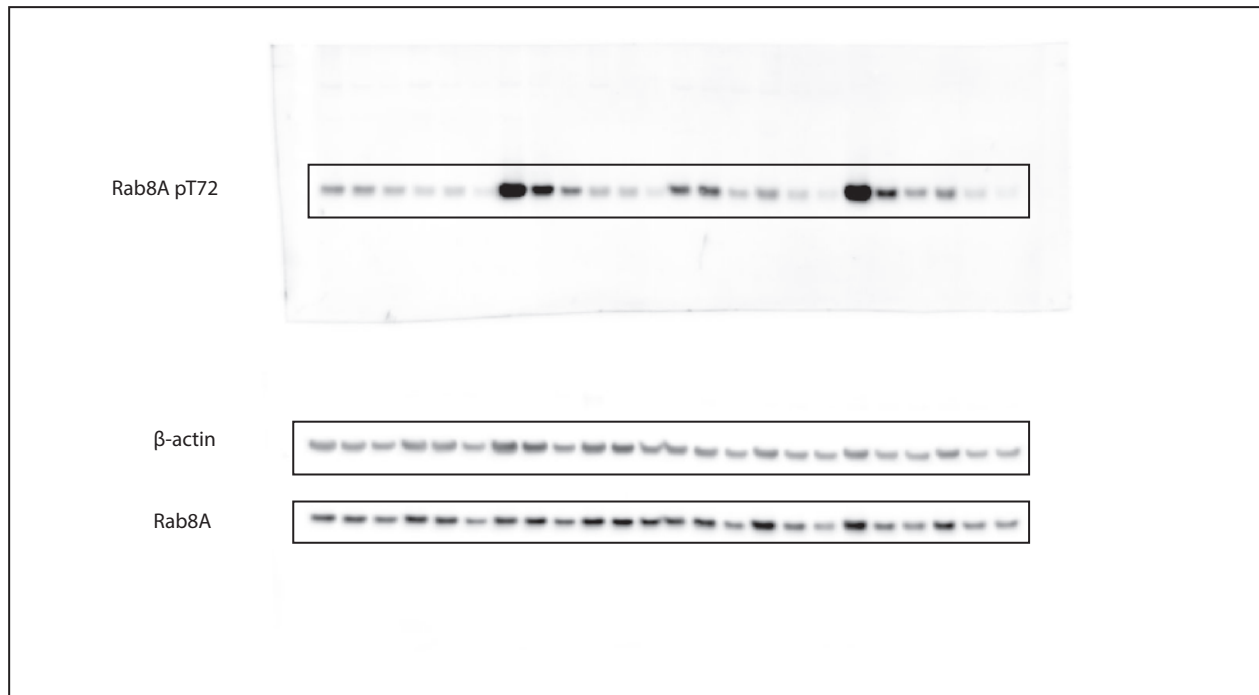

Supplement: Supplementary file 9 — Source Data for Figure 7 [file EMBJ-39-e104494-s007.pdf]
